# Simulation of the Spectrum Response for the THz Atmosphere Limb Sounder (TALIS)

**DOI:** 10.3390/s20020498

**Published:** 2020-01-15

**Authors:** Yongqiang Duan, Zhenzhan Wang, Haowen Xu, Wenyu Wang

**Affiliations:** 1 Key Laboratory of Microwave Remote Sensing, National Space Science Center, Chinese Academy of Sciences, Beijing 100190, China; duanyongqiang15@mails.ucas.ac.cn (Y.D.); xuhaowen16@mails.ucas.ac.cn (H.X.); wangwenyu14@mails.ucas.ac.cn (W.W.); 2 University of Chinese Academy of Sciences, Beijing 100094, China

**Keywords:** THz atmosphere limb sounder (TALIS), FFT spectrometer, simulation model, performance evaluation, calibration, sideband imbalance, quantization error

## Abstract

The THz atmospheric limb sounder (TALIS) is a microwave radiometer developed by the National Space Science Center of the Chinese Academy of Sciences for the detection of atmospheric trace gases. The observation range of the instrument mainly focuses on the middle and upper atmosphere (10–100 km above the earth’s surface). The detection targets include the temperature, pressure, and more than 10 kinds of atmospheric components. Its scientific goal is to improve our comprehension of atmospheric chemical composition and dynamics, and to monitor environmental pollution and sources in the atmosphere. The TALIS instrument is composed of an antenna, superheterodyne radiometers, and digital fast Fourier transform (FFT) spectrometers. By measuring the atmospheric thermal radiance in the wide frequency band with 118, 190, 240, and 643 GHz as the center frequency, the required volume mixing ratio (VMR) of atmospheric chemical species can be obtained. This paper introduces the characteristics of the TALIS instrument, and establishes a simulation model for the TALIS spectrometer. Through a joint simulation with an atmosphere radiative transfer simulator (ARTS), the TALIS instrument performance is evaluated from the aspects of calibration, the imbalance of two sidebands, the spectrum resolution, and quantization. The simulation results show that the two-point calibration can well-restore the radiance spectrum of the scene target and remove the influence of the spectral response function (SRF); the double side band (DSB) receiver with a 2 MHz resolution can meet the sensitivity and spectrum resolution requirements. Finally, the sensitivity errors of different quantization bits are given by the simulation and the results show that at 8-bit, the sensitivity and its degradation ratio are 1.251 K and 1.036 at a 2 MHz spectrum resolution and 100 ms integration time, respectively.

## 1. Introduction

High-precision earth atmosphere observations are the foundation of numerical weather forecasting and climate change research. Satellites can provide global atmospheric monitoring in a short time. The THz limb sounder can not only provide a better vertical resolution, but also collect chemical composition information in a wide range of height, which is not affected by the day and night cycle [1]. Limb sounding is a particularly useful technique in stratospheric and mesospheric temperature and chemistry detection, and has great potential in global wind measurement of the middle and upper atmosphere [2]. Microwave limb sounding remotely measures atmospheric parameters by observing millimeter- and submillimeter-wavelength thermal emission as the instrument field of view (FOV) is scanned through the atmospheric limb from above.

In the past two decades, several satellites have been launched, which were equipped with the payload of a limb sounder. Their observation data enable people to better understand the physical and chemical processes in the earth’s atmosphere. The microwave limb sounder (MLS) on the upper atmosphere research satellite (UARS) was the first limb sounder used in the microwave frequency band in space [3]. UARS-MLS includes three frequency bands: 63, 183, and 205 GHz. The system noise temperature is 400, 900, and 990–1530 K, respectively. A complete limb scan and calibration time of the instrument is 65 s, with an integration time of 1.8 s, and the vertical detection range is 5–95 km. UARS-MLS uses a Schottky diode mixer to down-convert the received signal. After the intermediate frequency (IF) signal is amplified, it is further down-converted to six spectral bands, and then enters six filter banks. The center frequency of each spectral band is 400 MHz, the bandwidth is 510 MHz, and the spectrum resolution is 2–128 MHz [4,5,6]. The Aura satellite is an atmospheric composition and environmental monitoring satellite of the earth observation system (EOS) of the United States. As the successor of UARS-MLS, EOS-MLS consists of five radiometers with center frequencies of 118, 190, 240, and 643 GHz and 2.5 THz, with more and higher detection frequency bands. The integration time is 0.16 s, and the vertical detection range is 0–90 km. The 118 GHz radiometer is designed as a single sideband (SSB) with system noise of 1000–1600 K; others are a double sideband (DSB) with a noise temperature of 900–18,000 K. The backend includes four kinds of spectrometers (12 wideband filter banks, five medium bandwidth filter banks, 19 standard filter banks, and four autocorrelation spectrometers), which adopt different spectrum resolutions and bandwidths to cover different height ranges, and the spectrum resolution is 0.15–500 MHz [7,8,9]. Odin is a joint mission satellite of upper atmospheric physics and astronomy, which includes a sub-millimeter radiometer (SMR) [10]. Odin-SMR was the first limb sounding system to be cooled by a constant temperature refrigerator in space. The center frequencies of SMR are 119, 495, and 561 GHz, respectively, and the system noise temperatures are 600, 3300, and 3300 K, respectively. The integration time is 0.875 to 3.5 s, the sensitivity is 1 K when the spectrum resolution is 1 MHz, and the vertical detection range is 15–120 km. The backend is composed of two autocorrelation spectrometers and one acoustooptic spectrometer, with a spectrum resolution of 0.1–1 MHz [11,12,13]. The superconducting submillimeter-wave limb-emission sounder (JEM/SMILES) was launched by Japan in 2009. It is loaded in the Japanese experimental cabin of the international space station and mainly used for the detection of the atmospheric radiance signal in the 643 GHz band [14]. JEM/SMILES was the first to use a superconductor insulation superconductor (SIS) mixer and 4 K mechanical refrigeration thermostat to reduce system noise in space. It has three detection frequency bands, the center frequency is concentrated at 643 GHz, the system integration time is 0.5 s, and the sensitivity is 0.7–1 K [15,16]. The backend is composed of two acoustooptic spectrometers, with a bandwidth of 1.2 GHz and a spectrum resolution of 1.8 MHz [17,18]. Apart from space-borne limb sounders, there are also some airborne limb sounders. The Terahertz and SubMMW limb sounder (TELIS) instrument is a cryogenic three-channel DSB spectrometer with an integration time of 1.5 s for limb sounding of stratospheric trace gases. The frequency ranges are 497–504, 480–650, and 1750–1890 GHz, respectively, and the system noise are 2000, 200, 3000–4000 K, respectively. A digital autocorrelation spectrometer with 4 GHz bandwidth and 2 MHz resolution serves as backend shared among the channels [19]. The airborne microwave stratospheric observing system (AMSOS) is a radiometer for stratospheric water vapor measurements at 183.3 GHz. It is a heterodyne receiver using a subharmonic mixer to convert the signal down to an IF of 3.7 GHz. A narrowband FFT spectrometer with a resolution of 12 kHz over a 25 MHz bandwidth and a broadband one with 61 KHz resolution over a 1 GHz bandwidth with an integration time of 2.8 s are used as backend [20].

In addition to UARS-MLS, EOS-MLS, Odin-SMR, and JEM/SMILES, some new space-borne microwave limb sounders are also being developed. The new generation of earth atmosphere satellites proposed by NASA includes the scanning microwave limb sounder (SMLS), working in frequency bands of 240 and 643 GHz [21]. The superconducting submillimeter wave limb sounder 2 (SMILES-2) proposed by the Japan Aerospace Exploration Agency will scan the atmosphere from the low stratosphere to the low thermosphere to obtain a temperature profile of 15 to 160 km [22,23], with a system noise temperature from 120 to 990 K [1]. Stratospheric inferential wind (SIW) is a small submillimeter wave limb sounder developed in Sweden, which is mainly used to measure wind, temperature, and trace gases in the stratosphere. Its superheterodyne radiometer works with a DSB receiver, with the system noise of 1000–1200 K. The bandwidth of the backend autocorrelation spectrometer of SIW is 8 GHz, and the spectrum resolution is 1 MHz [24].

The THz atmosphere limb sounder (TALIS) is a radiometer being designed by the National Space Science Center of the Chinese Academy of Sciences for the high precision measurement of atmospheric temperature and key chemical species. The radiometer consists of four frequency bands of 118, 190, 240, and 643 GHz, similar to EOS-MLS. Its backend digital spectrometer consists of 22 FFT spectrometers. Each spectrometer has a bandwidth of 2 GHz with a spectrum resolution of 2 MHz and a quantization level of 8-bit. The specific target of TALIS is to provide information for the study of the dynamics and chemistry of the middle and upper atmosphere by measuring the temperature, volume mixing ratio (VMR) profile of atmospheric species, and cirrus with a higher spectrum resolution. Compared with the previous loads, TALIS will use the FFT spectrometer based on a field programmable gate array (FGPA) for the first time, which will greatly improve the spectrum resolution, measurement accuracy, and instrument stability.

In this paper, Section 2 describes the instrument characteristics and system specification; the simulation model of TALIS is established in Section 3, and then, through a joint simulation with an atmosphere radiative transfer simulator (ARTS), the preliminary performance of the instrument is evaluated in Section 4 from four aspects: System calibration, imbalance between two DSB sidebands, spectrum resolution, and quantization error. Section 5 is the summary of this paper.

## 2. Instrument Characteristics

**Figure 1:**
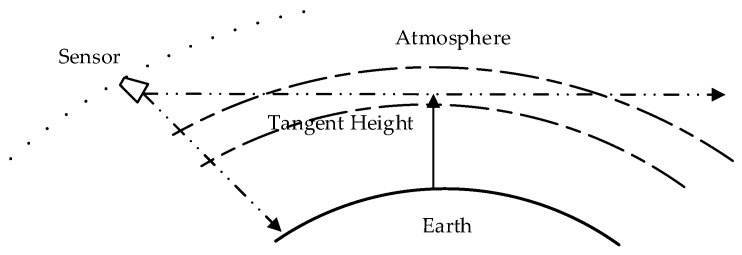
Schematic diagram of limb sounding.

A limb sounder remotely measures atmospheric parameters by observing thermal emission as the instrument scanning through the atmospheric limb. Figure 1 shows the principle of limb sounding [25]. The TALIS instrument will be set at a sun-synchronous orbit at a normal altitude of 600 km. The antenna will scan the limb vertically from the surface to about 100 km. During the scanning, the corresponding earth circle angle between the observation point and the tangent point is about 66–68°. The antenna samples at a constant speed in the range of 0–2.6°, the sampling interval is about 1 km, the integration time of each sampling point is 100 ms, and the total observation time of the antenna in the scene target is 10 s. Two blackbodies are used as the cold and hot targets, and the cold spaces are used as extra targets to estimate the antenna effect and nonlinearity. TALIS will first view the hot target and the cold target in 3 s, and then scan the limb from 0 to 100 km in 10 s. Finally, it will view the cold space at 200 km in 5 s. The process of retracing is the same. Assuming the response function of the instrument is constant within 36 s, the TALIS instrument can be calibrated by the cold and hot target to obtain the radiance spectrum of the scene target at each height.

The TALIS instrument mainly consists of an antenna, receivers, and spectrometers. The antenna is composed of a single reflector and four frequency-independent feeds. The aperture of the reflector is 1.6 m, the focal diameter ratio is 0.75, and the angle between the incident direction of the electromagnetic wave and the horizontal line (tangent direction of the satellite forward) is 21–23°. The TALIS receiver is composed of four superheterodyne radiometers working at ambient temperature, with the frequency bands of 118, 190, 240, and 643 GHz, respectively. Table 1 gives the band range of each receiver. The 118 GHz radiometer, covering the strong O_2_ line at 118.75 GHz, is used to measure the atmospheric temperature and tangent pressure. The 190 GHz radiometer is mainly used to measure ClO, N_2_O, H_2_O, etc. The 240 GHz radiometer is used to measure the O_3_ and CO absorption. In addition, the atmospheric absorption caused by water vapor at the top of troposphere in this band is small enough to effectively measure the ozone distribution at the top of the troposphere. The 643 GHz radiometer can measure the absorption lines of the lowest frequency of HCl; the strongest absorption lines of ClO; and the weak absorption lines of BrO, HO_2_ and N_2_O [2]. The proposed characteristics of the TALIS instrument, which may be slightly modified in the future, are summarized in Table 1. The system noise for the four radiometers is better than 1000, 1000, 1000, and 2300 K, respectively. The IF bandwidth and spectrum resolution of all spectrometers are 2 GHz and 2 MHz, respectively.

**Figure 2:**
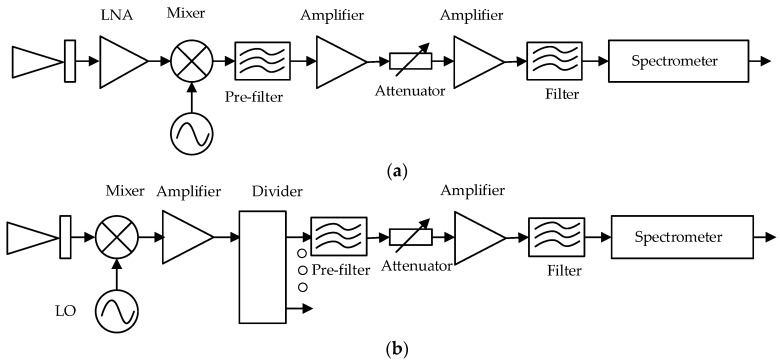
The block diagram of the TALIS instrument: (**a**) 118 GHz; (**b**) other bands.

According to the different positions of low noise amplifier (LNA) and the mixer of the RF receiver, TALIS adopts two kinds of receivers: The 118 GHz radiometer adopts amplification first and then mixing, and the block diagram is shown in Figure 2a. After the LNA, the mixer and filters are used. The other three bands have different structures, which is shown in Figure 2b. Since there is no LNA in these bands at present, the three frequencies are mixed first and then amplified.

The sensitivity of the receiver depends on the noise temperature of the system, which is also related to the integration time and spectrum resolution (or sub-channel bandwidth). Under the integration time of 100 ms and system noise temperature of 1000, 1000, 1000, and 2300 K, the theoretical sensitivities of the four bands are 2.2, 2.2, 2.2, and 5.1 K, respectively, as listed in Table 1.

With the increasing requirements of the input bandwidth and spectrum resolution, the traditional spectrometer is unable to meet the requirements. The digital spectrometer has the advantages of a large bandwidth and configurable spectrum resolution. In addition, it has a strong anti-interference ability, high stability, and small size, and can be used more flexibly [26]. There are several types of digital spectrometer, including the FFT spectrometer and autocorrelation spectrometer. The autocorrelation spectrometer needs a number of multiplications of N×(N−1) (*N* is the number of data points), while the FFT spectrometer only needs a number of 0.5Nlog(2N), which greatly reduces the amount of calculations required [26]. Therefore, the backend of TALIS adopts the FFT spectrometer. The power spectrum of the scene target is obtained by squaring the spectrum of the output signal of the receiver. Since the spectral response function (SRF) is not flat in the band, the power spectrum is weighted SRF. The signal of the target is down-converted from the initial band of $f_{1}$ to $f_{2}$ to an IF band from 0 to $f_{2}-f_{1}$. The IF band is sampled periodically at a rate of up to 4 GS/s. Therefore, the IF bandwidth $f_{2}-f_{1}$ is 2 GHz. In order to record the IF signal from 0 to 2 GHz, the signal must be sampled every 0.5 ns at least. We can use 2048-point FFT to generate 1024 channels. The time needed to obtain these data is 1.024 us.

## 3. Simulation Model of the TALIS Spectrometer

**Figure 3:**
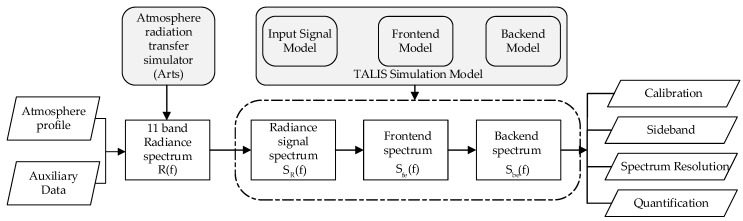
Simulation flow chart for performance evaluation of TALIS.

In order to preliminarily evaluate the performance of the TALIS instrument, we developed an instrument simulation model, which can simulate the working process and characteristics of TALIS in combination with ARTS. The whole simulation and evaluation process is shown in Figure 3. Firstly, the radiance of each frequency band and tangent height is generated by ARTS, which needs the atmosphere profile and other auxiliary data as the input. Then, the radiance is used as the input of the TALIS simulation model to generate the radiance signal, frontend signal, and backend power spectrum, from which we can evaluate the instrument performance. This section describes two main parts for the simulation system: ARTS and TALIS simulation model. In the next section, the calibration, imbalance between two DSB sidebands, spectrum resolution, and quantization error of TALIS are simulated and analyzed.

### 3.1. Generating Radiance by ARTS

ARTS is a comprehensive radiative transfer model established by Bremen University and Chalmers University for the research of satellite atmospheric science. It has been gradually improved in subsequent development, and has been widely used in the radiative transfer simulation of satellite remote sensing technology in the way of nadir observation, limb sounding, and occultation observation [27]. At the same time, ARTS can be used in physical quantities, such as atmospheric transmittance, scattering, and transmission radiance, as well as the solar radiance reflected from the surface. ARTS use the line-by-line integration mode to calculate the radiative transfer equation, which has a very high accuracy.

**Figure 4:**
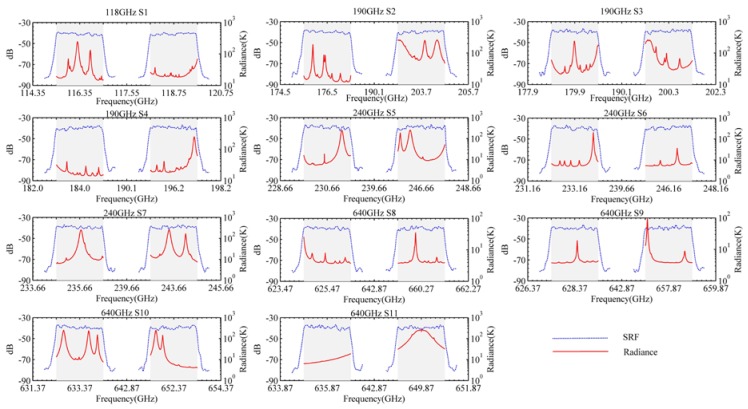
The spectral response functions (SRFs) of 11 bands of TALIS and the atmospheric radiance spectrum at the tangent height of 30 km. The red curve represents atmosphere radiance and the blue dotted line represents SRF.

In this paper, ARTS 2.3 is used to simulate the atmospheric radiances of the tangent height for TALIS, which are used as the input of the TALIS simulation model. The instrument frequency setting follows the TALIS characteristics described in Table 1. The ideal rectangular SRF is used in ARTS. Radiances of 11 bands of TALIS are generated, ranging from 10 to 90 km, and the interval is 10 km. A typical mid-latitude summer atmospheric condition extracted from fast atmospheric signature code (FASCOD) is chosen to perform the simulation. The atmospheric profiles include the temperature, O_3_, HCl, ClO, N_2_O, NO, NO_2_, HOCl, H_2_O, HNO_3_, HCN, CO, SO_2_, BrO, HO_2_, H_2_CO, and CH_3_Cl. The high-resolution transmission (HITRAN) database is used for line-by-line absorption calculation. The red curve in Figure 4 shows the simulated radiance spectrum of 11 bands of TALIS at a tangent height of 30 km.

### 3.2. TALIS Simulation Model

The TALIS instrument mainly consists of two parts: The RF frontend and digital backend. In addition, it needs a thermal radiance noise signal as the input. Therefore, the simulation model for the TALIS spectrometer can be divided into three parts: The thermal radiance noise signal model, RF frontend model, and digital backend spectrum sampling model.

• Thermal Noise Signal Model

The radiometer receives the thermal noise of the target, and the radiance represents the energy of the thermal noise. The purpose of thermal noise modeling is to transform the radiance generated by ARTS into the thermal noise signal entering the radiometer. In general, we use white Gauss noise (WGN) to represent thermal noise, and WGN obeys a normal distribution with a mean of zero and variance of *σ*. Therefore, thermal noise modeling is performed to determine the value of *σ*. The relation between *σ* and radiance *R* is given by [28] as

(1) $$\sigma^{2}=\frac{kR}{2},$$

where *k* is the Boltzmann constant. A set of time domain noise signals s[i] with a radiance of *R* can be generated by

(2) $$s[i]∼N(\mu ,\sigma^{2}),$$

where *N* represents the normal distribution, the mean is equal to 0, and the variance is determined by Equation (1). *I* is the number of FFT points. Equation (2) can be used to model the thermal noise of the hot target and cold target with a uniform radiance spectrum. However, in order to generate thermal noise of the scene target with a nonuniform radiance spectrum, the equation needs to be further improved.

Firstly, we need to generate a reference noise signal $s_{ref}[i]$ with a radiance of $R_{ref}$. The spectrum of the signal $S_{ref}[i]$ can be obtained by the *i*-point Fourier transform and can be expressed as

(3) $$S_{ref}[i]∼N(0,\sigma_{ref}{}^{2}/\pi^{2}),$$

where $\sigma_{ref}{}^{2}$ can be obtained by Equation (1). According to the properties of a normal distribution, the spectrum of noise signal $S_{R(f)}[i]$ with radiance spectrum R(f) can be obtained by dividing $\sqrt{R_{ref}}$ at each point and multiplying by $\sqrt{R(f)}$:

(4) $$S_{R(f)}[i]=S_{ref}[i]\cdot \sqrt{R(f)/R_{ref}}$$

**Figure 5:**
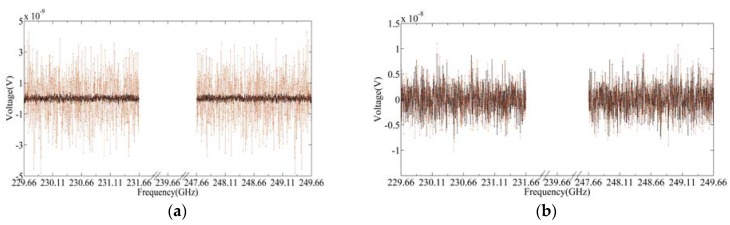
Thermal noise signal spectrum for the hot target and cold target in 240 GHz S5 upper and lower sidebands: (**a**) *Tsys* = 0 K; (**b**) *Tsys* = 1000 K.

**Figure 6:**
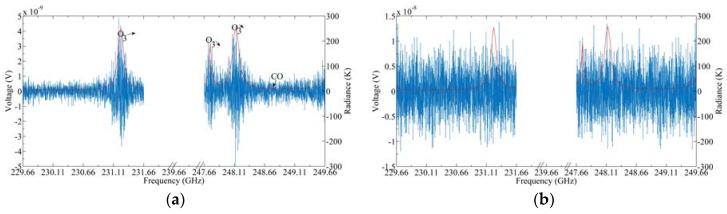
Thermal noise signal spectrum for the scene target in 240 GHz S5 upper and lower sidebands: (**a**) *Tsys* = 0 K; (**b**) *Tsys* = 1000 K.

Figure 5 and Figure 6 show the thermal noise simulation results of the upper and lower bands of 240 GHz S5 by Equations (2) and (4), respectively, where Figure 5a and Figure 6a are the results of system noise of 0 K, and Figure 5b and Figure 6b are those at system noise of 1000 K. The red curve in Figure 6 represents the radiance which is used as the input of the thermal noise model. It can be seen from Figure 6a that the shape of the thermal noise spectrum is basically the same as that of the input radiance spectrum. However, when the system noise is 1000 K, the shape of the target radiance spectrum is difficult to distinguish. Therefore, it is necessary to average this noise signal at enough intervals to obtain the scene target radiance spectrum, which is based on the requirement of sensitivity.

• Frontend Model

The frontend of TALIS is defined as the whole RF part. The channel SRF can be used to represent the frontend transfer function. Since the receiver of TALIS is DSB, the SRFs of both the upper sideband and lower sideband are needed. It should be noted that the frontend frequency of TALIS is very high. Limited by computer resources, we can simulate the mixing process directly in the IF band. The requirement of the in-band flatness of TALIS is better than 3 dB, and the IF bandwidth is 2 GHz. The simulated spectrum of 11 bands of SRFs with an in-band flatness of 3 dB are shown in the blue dotted line in Figure 4, in which the upper and lower sidebands are assumed symmetrically. The signal spectrum $S_{fe}$ after the frontend can be expressed as

(5) $$S_{fe}=(S_{ru}\cdot SRF_{u}+flip(S_{rl}\cdot SRF_{l})),$$

where $S_{ru}$ and $S_{rl}$ are radiances of the upper sideband and lower sideband, respectively. $SRF_{u}$ and $SRF_{l}$ are SRFs of the upper sideband and lower sideband, respectively. *Flip* represents inversion of an array, because in a DSB receiver, the lower sideband will *flip* in the frequency domain when mixing. $S_{fe}$ is the spectrum of the frontend output signal. The output signal of the frontend $s_{fe}$ can be obtained by the *i*-point inverse Fourier transform of $S_{fe}$.

**Figure 7:**
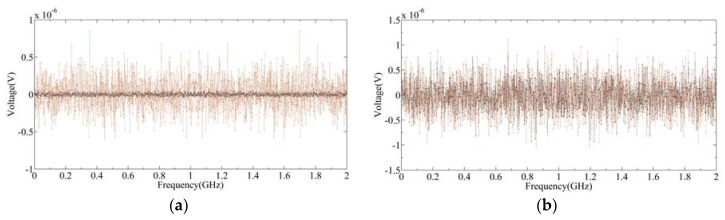
Signal spectrum of cold and hot targets at the frontend of the 240 GHz S5 spectrometer, where the red dot represents the hot target and black dot represents the cold target: (**a**) *Tsys* = 0 K; (**b**) *Tsys* = 1000 K.

**Figure 8:**
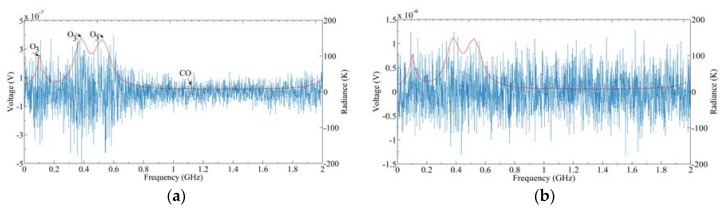
Signal spectrum of the scene target in front of the 240 GHz S5 spectrometer: (**a**) *Tsys* = 0 K; (**b**) *Tsys* = 1000 K.

Figure 7a,b are the frontend output signal spectrum of the hot target and cold target when the system noise is 0 and 1000 K, respectively. Figure 8a,b show the frontend output signal spectrum of the scene target when the receiver noise is set at 0 and 1000 K, respectively, and the red curve represents the DSB radiance spectrum. It can be seen from the figure that after mixing, the bandwidth of the IF signal is 2 GHz, and the radiation spectra of the upper and lower sidebands are overlapped. In addition, when the receiver noise is 1000 K, the radiance spectrum of the scene target is hard to distinguish.

• Digital Backend Model

The backend of each radiometer in TALIS is one or several FFT spectrometers, which are used to calculate the power spectrum of the frontend output signal. Due to the linear relationship between the power and the radiance, the radiance spectrum can be expressed by the output power spectrum. Backend modeling is performed to simulate the working process of the FFT spectrometer. The process includes sampling, quantization, windowing, Fourier transforming, and integration of the frontend output signal.

The relationships between the sampling rate *fs*, IF bandwidth $B_{IF}$, number of FFT points (or channel number) *I*, spectrum resolution Δf, and integration time τ need to be explained here. First of all, the *fs* of the simulation system is twice that of $B_{IF}$ and Δf is equal to *fs* divided by *i*. When *I* is determined, the number of time-domain signals for the Fourier transform is determined, which is also equal to *I*, so that the limited integration time and FPGA resources can be used to the greatest extent. Taking 240 GHz S5 as an example, the IF bandwidth is 2 GHz, so the sampling rate is set to 4 GS/s. The spectrum resolution is about 2 MHz. Therefore, the number of FFT points is set to 1024, and the number of time domain signals for each FFT transformation is also 1024. The integration time for one time series is 1/fs × 1024. Therefore, in order to achieve the integration time of 100 ms, it is necessary to average the power spectrum of 400,000 cycles calculated by FFT.

The frontend signal is quantized to *n*-bit to get the quantization signal:

(6) $${\hat{s}}_{fe}[i]=Q_{n}[s_{fe}[i]],$$

where $Q_{n}$ represents *n*-bit quantization for the frontend signal.

After quantization, the output power spectrum of the backend can be obtained by squaring the spectrum of the output signal of the RF frontend. The calculation of the power spectrum of the backend signal can be expressed as follows [29]:

(7) $$S_{be}[i]={\left|FFT({\hat{s}}_{fe}[i])\right|}^{2}.$$

**Figure 9:**
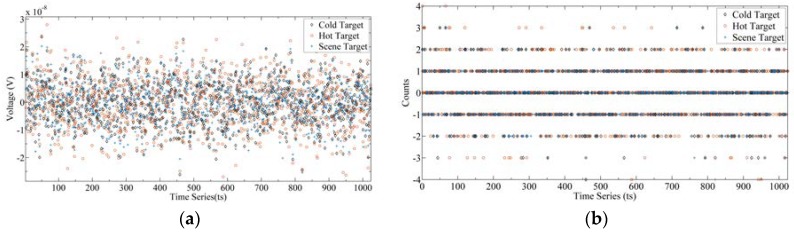
Frontend output signal of the cold target, hot target, and scene target: (**a**) output analog signals; (**b**) output signals after 3-bit quantization.

**Figure 10:**
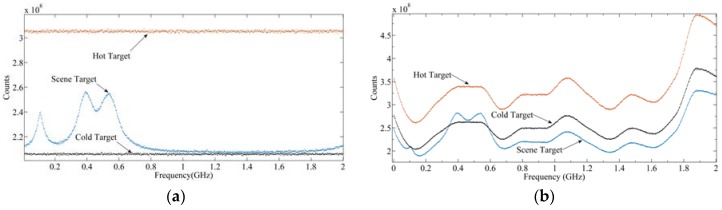
The output signal of the backend under the integration time of 100 ms: (**a**) SRF is a rectangular function; (**b**) SRF is not ideal.

It should be noted that since the RF frontend SRF is not a rectangular function, it will affect the shape of the output power spectrum. The output radiance spectrum can only be obtained through calibration, which will be detailed in Section 4.1. Figure 9a shows the output signal of the frontend, and Figure 9b shows the result after 3 bit quantization. The black diamonds, red circle, and blue cross represent the output signals of the cold target, hot target, and scene target, respectively. It can be seen from Figure 9b that after 3-bit quantization, the amplitude of the signal becomes a discrete value. Figure 10 is the power spectrum calculated by Equation (7), where Figure 10a is the result of SRF with an ideal rectangular function, and Figure 10b is the result of SRF with 3 dB fluctuation. It can be seen that due to the influence of SRF, the power spectrum of the spectrometer is no longer an ideal shape of the radiance spectrum. Therefore, calibration of the power spectrum is needed.

**Figure 11:**
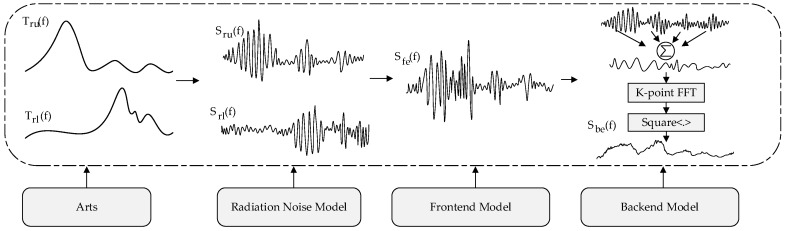
Simulation modeling steps.

Summarily, the process of simulating the spectrum response of TALIS is shown in Figure 11, including the following four steps:

Step 1: Generating 11 bands of the radiance spectrum from 10 to 90 km through ARTS;
Step 2: Modeling the thermal noise signal of hot and cold targets by Equation (2), and modeling the scene target signal by Equation (4). The system noise of each frequency band is shown in Table 1, and the scene target radiance spectrum is obtained from Step 1;
Step 3: Modeling the frontend output signals corresponding to the hot and cold targets and the scene targets, respectively, through Equation (5);
Step 4: The process of backend quantization and power spectral density (PSD) calculation is represented by Equations (6) and (7), respectively. In addition, by averaging 400,000 PSD, the PSD with the expected integration time of 100 ms can be obtained.

## 4. Simulation Results of the TALIS Instrument Performance

### 4.1. Calibration

The traditional total power radiometer uses two-point calibration to determine the scene target radiance. The calibration principle of the spectrometer is similar. The difference is that it needs to calibrate each channel using two-point calibration to obtain the radiance of each channel [30,31]. The radiance spectrum can be obtained by connecting the radiance of all channels. Early spectrometers obtain several channels through filter banks and then use two-point calibration for each channel. The principle of this is exactly the same as that of the total power radiometer. The FFT spectrometer or autocorrelation spectrometer processes the frontend signal through FPGA, and obtains the power spectrum. The output of the FFT spectrometer is the power of discrete frequency points. Therefore, the calibration method of the FFT spectrometer is similar to that of the filter bank spectrometer. That is to say, the power of each frequency point is calibrated by two-point calibration through the known radiance of the hot target and cold target. TALIS scans the cold target, hot target, and scene target in turn. The radiance of the scene target is calculated by two-point calibration, through which the influence caused by SRF in Figure 10b can be eliminated.

**Figure 12:**
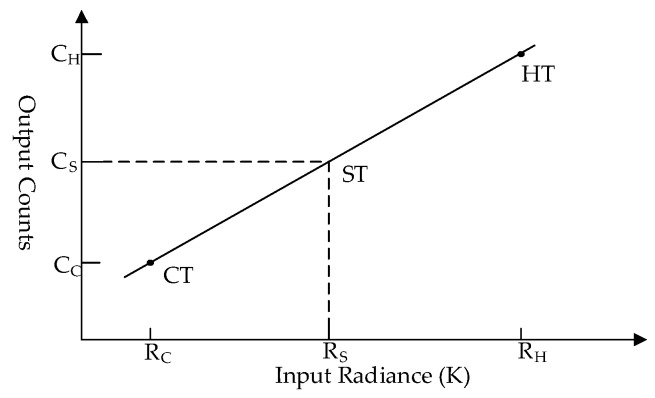
Linear relationship between counts and radiance.

The principle of two-point calibration is illustrated in Figure 12. Here, *R* is the radiance entering the radiometer and *C* is the count of the backend power spectrum. The calibration equation needs to be determined for each channel, which can be expressed as

(8) $$C_{i}=a_{i}\cdot R_{i}+b_{i},$$

where *i* represents the *i*th channel, and $a_{i}$ and $b_{i}$ are the primary term and constant term of the *i*th calibration equation, which can be obtained from

(9) $$\{\begin{array}{c} a_{i}=\frac{C_{H,i}-C_{C,i}}{R_{H,i}-R_{C,i}}\\ b_{i}=\frac{C_{C,i}R_{H,i}-C_{H,i}R_{C,i}}{R_{H,i}-R_{C,i}} \end{array}.$$

In the simulation model, 3 and 290 K targets which have uniform radiance in the 2 GHz bandwidth are used as cold and hot targets, respectively. The radiance spectrum of the scene target at 20, 30, and 40 km is used as the input radiance. It is assumed that the instrument is stable in a calibration cycle.

**Figure 13:**
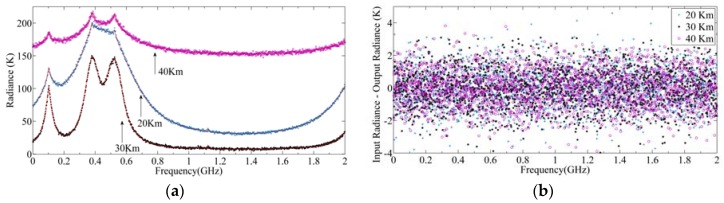
Radiance spectrum of the scene target at a 20, 30, 40 km height of 240 GHz S5: (**a**) Output radiance spectrum of three heights; (**b**) the difference between input radiance and output radiance.

The scene target power spectrum is calibrated by the output power spectrum of the cold and hot targets. The calibration results are shown in Figure 13a. Here, the red curve represents the input radiance spectrum of the simulation model, and the purple, blue, and black dots are the output radiance spectrum of 20, 30, and 40 km, respectively, after calibration. It can be seen that the output radiance spectrum is completely consistent with the input radiance spectrum, that is, the effect of SRF is eliminated through calibration.

For a spectrometer, sensitivity is the most important indicator. The definition of sensitivity for a spectrometer is similar to that of a total power radiometer, which can be defined as the minimum resolvable input radiance of a spectrometer in the frequency domain. The sensitivity of spectrometer $\Delta R_{f}$ can be obtained by standard deviation analysis [32], which can be expressed as

(10) $$\Delta R_{f}={STD(R_{out}(i)-R_{in}(i))|}_{i=1:m},$$

where $R_{out}$ represents the output radiance spectrum and $R_{in}$ is the input radiance spectrum. Figure 13b shows the difference between $R_{out}$ and $R_{in}$. The means of difference for targets of 20, 30, and 40 km are −0.029, 0.012, and −0.038 K, respectively, and the standard deviations of those are 1.146, 1.150, and 1.134 K, respectively. The theoretical and simulation values of the means and standard deviation under different integration times are given in Table 2, from which it can be seen that the simulation value is very close to the theoretical value. It also verifies the correctness of the TALIS simulation model.

### 4.2. Imbalance between Two DSB Sidebands

DSB and SSB are two kinds of receivers commonly used in radiometers. In an SSB system, the receiver only receives one sideband signal, so it can keep an independent spectrum. DSB combines the upper and lower sidebands into one sideband, so the bandwidth of DSB is twice that of SSB under the same IF bandwidth. For the spectrometer, on the one hand, the channel of DSB contains more lines of trace gas, and the sensitivity is better than SSB, which will help us to retrieve more trace gases. On the other hand, the lines of these trace gases would be superimposed together, which would make some lines difficult to distinguish from the others and may be detrimental to the retrieval accuracy for those species.

**Figure 14:**
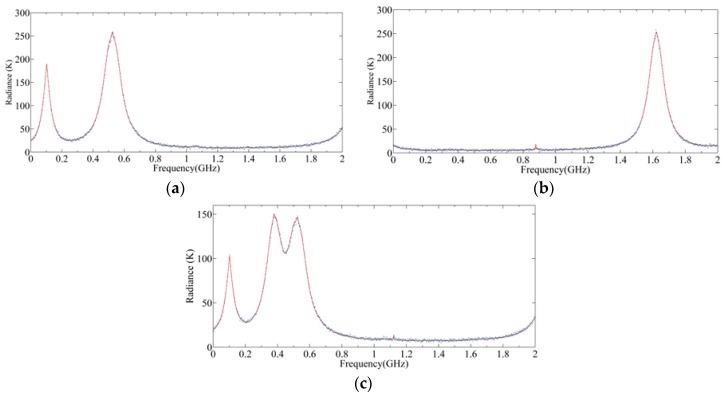
Radiance spectrum of single sideband (SSB) and double sideband (DSB): (**a**) Upper sideband; (**b**) lower sideband; (**c**) DSB.

Figure 14a,b show the radiance of the upper and lower sidebands of 240 GHz S5 using SSB, respectively, and Figure 14c shows the radiance of the mixed sidebands of 240 GHz S5 using DSB. The red curves represent the input radiance and the blue dots represent the output radiance. Generally speaking, a DSB system can keep all the radiance spectrum information of the two sidebands of SSB, so these gas contents can be retrieved according to the output radiance spectrum of DSB. However, in some areas, such as the CO line, the line is obviously weakened due to the superposition of upper radiance. At 0.42 GHz, the two O_3_ lines are overlapped, which may affect the retrieval precision.

According to the receiver configuration of TALIS, the frontend of 118 GHz is LNA, while the frontends of other frequency bands are mixers. With no amplifier to prefilter the signal, the radiometer of other frequency bands can only use the DSB receiver. For the 118 GHz radiometer, although LNA is used, its gain is too low to suppress the noise in the imagery frequency band, so we decided to use the DSB receiver too.

After deciding to use the DSB system for all radiometers, we needed to consider the influence of imbalance between two DSB sidebands. Equations (11) and (12) represent the theoretical values of the input and output radiance of a DSB receiver, respectively:

(11) $$R_{in}(i)=\frac{R_{u}(i)+R_{l}(i)}{2}$$

(12) $$R_{out}(i)=\frac{R_{u}(i)\cdot SRF_{u}(i)+R_{l}(i)\cdot SRF_{l}(i)}{SRF_{u}(i)+SRF_{l}(i)},$$

where $R_{u}(i)$ and $R_{l}(i)$ are the radiances of upper sideband and lower sideband, respectively, and $SRF_{u}(i)$ and $SRF_{l}(i)$ are SRFs of the upper sideband and lower sideband, respectively. It can be seen from Equation (12) that when there are some imbalances between $SRF_{u}(i)$ and $SRF_{l}(i)$, the output radiance of the channel will be deviated. Therefore, the relationship between this imbalance and radiance error needs to be evaluated. The imbalance between two DSB sidebands is defined as $\left|SRF_{u}(i)-SRF_{l}(i)\right|/SRF_{l}(i)$.

**Figure 15:**
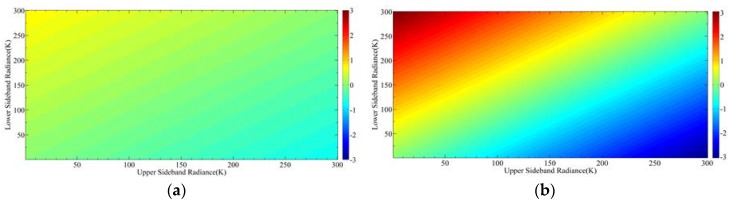
Radiance error caused by the imbalance of two DSB sidebands: (**a**) Imbalance is 0.5%; (**b**) imbalance is 2%.

Figure 15a,b show the simulation results of radiance error when the imbalance is 0.5% and 2%, respectively. The horizontal axis and vertical axis represent the radiance of the upper sideband and lower sideband, respectively. First of all, it can be seen that the larger the radiance difference between the upper and lower sidebands, the larger the error caused by the sideband imbalance. Secondly, when the imbalance is 0.5%, the maximum radiance error is 0.6 K, and when the imbalance is 2%, the maximum radiance error is 3 K, which means that the radiance error increases with the increase of imbalance.

**Figure 16:**
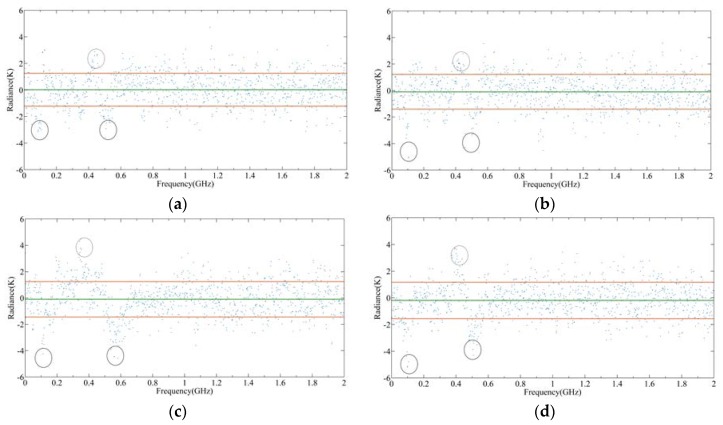
Simulation results of radiance error caused by the imbalance in 240 GHz S5: (**a**) Imbalance is 0.5%; (**b**) imbalance is 1%; (**c**) imbalance is 1.5%; (**d**) imbalance is 2%.

Figure 16 shows the radiance errors of 240 GHz S5 with different imbalances. The circles in each figure represent large radiance errors which are caused by imbalance. The red line indicates the range within 1 σ from the mean indicated by green line. Firstly, it can be seen that the errors at 0.1, 0.4, and 0.52 GHz are the largest because the O_3_ lines with high radiance are overlapped with low radiance when mixing. Secondly, when the imbalance is 0.5%, the maximum radiance error is about 0.6 K. However, it can be seen from Figure 16a that this error is submerged by measurement noise, thus it will have little influence on the output radiance. However, when the imbalance increases, the radiance error becomes larger and the influence on the output radiance becomes larger too. Therefore, it is very important for the spectrometer to measure the radiance spectrum accurately so that the upper and lower sideband channels exhibit a good sideband balance. In order to achieve a good radiance accuracy, the imbalance of TALIS should be better than 0.5%.

### 4.3. Spectrum Resolution

When the bandwidth of the spectrometer is determined, the number of channels is determined by the spectrum resolution. For a spectrometer, the spectrum resolution is a very important indicator which determines the narrowest line that can be detected. Theoretically, some narrower lines can be detected with a higher spectrum resolution. However, the higher the spectrum resolution, the narrower the channel bandwidth. From the sensitivity equation, the sensitivity will be worse. For some lines with small radiance, they may be submerged by noise. Therefore, the spectrum resolution and sensitivity are a pair of contradictory indicators. It is necessary to determine the spectrum resolution through simulation, so that both the spectrum resolution and sensitivity of the spectrometer can meet the requirements at the same time.

**Figure 17:**
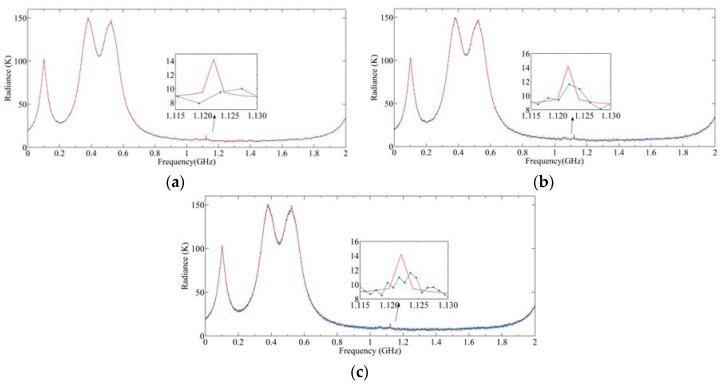
Output radiances under different spectrum resolutions: (**a**) 4 MHz; (**b**) 2 MHz; (**c**) 1 MHz.

Figure 17 shows the simulation results of 512, 1024, and 2048 channels, respectively, which correspond to the spectrum resolutions of 4, 2, and 1 MHz, respectively. It can be seen from Figure 17a that although the sensitivity of 4 MHz is the best, the CO line has been missed due to the fence effect. When the number of channels is 2048, the spectrum resolution is about 1 MHz, and all gas lines can be observed. However, the sensitivity is the worst at this time, which makes it difficult to distinguish some lines with small radiance. It can be seen from Figure 17c that the CO line may be submerged by the noise. When the channel number is 1024, as shown in Figure 17b, the spectrum resolution and sensitivity meet the requirements to distinguish the CO line simultaneously. Therefore, 1024 channels with a 2 MHz spectrum resolution is optimal.

**Figure 18:**
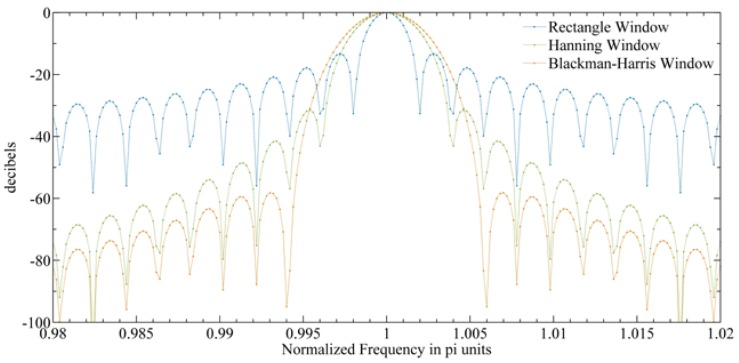
Three commonly used window functions for fast Fourier transform (FFT) weighting.

In addition, the spectrum leakage caused by FFT to the noise signal should also be considered [33]. The FFT algorithm assumes that the signal is periodic. If the signal is not periodic, it will lead to discontinuity at the interval of the time length and spectrum leakage will thus occur. This will introduce a wide range of frequencies in the frequency domain and cause the signal to expand to adjacent frequency bins. Figure 18 shows three commonly used window functions. If the rectangular window function is applied before FFT (i.e., without any processing), the first sidelobe attenuates by 13 dB relative to the main lobe, and the sidelobe drop rate is 6 dB per octave. Therefore, the selectivity of FFT is very poor, which will produce many ripples in the passband [33]. By reducing the discontinuity at both ends of time recording, that is, by multiplying the data with the appropriate window function, the effect of spectrum leakage can be limited [33]. Generally speaking, there is always a trade-off between the main lobe width and the side lobe leakage: With the decrease of the sidelobe level, the main lobe width increases. Therefore, when a rectangular window is used, the side lobe suppression is poor and the main lobe is narrow. This will cause the influence of the nearby channel on the channel to be smaller and the influence of the far channel on the channel to be greater. When the Hanning window or Blackman window is used, the main lobe is wider and the side lobe suppression is better, so the influence of the nearby channel on the channel is larger and that of the far channel is smaller. Sensitivity is the most important indicator. Therefore, under a certain spectrum resolution, we can use the above three window functions to calculate the sensitivity of the system, and choose the window function according to the results of sensitivity.

**Figure 19:**
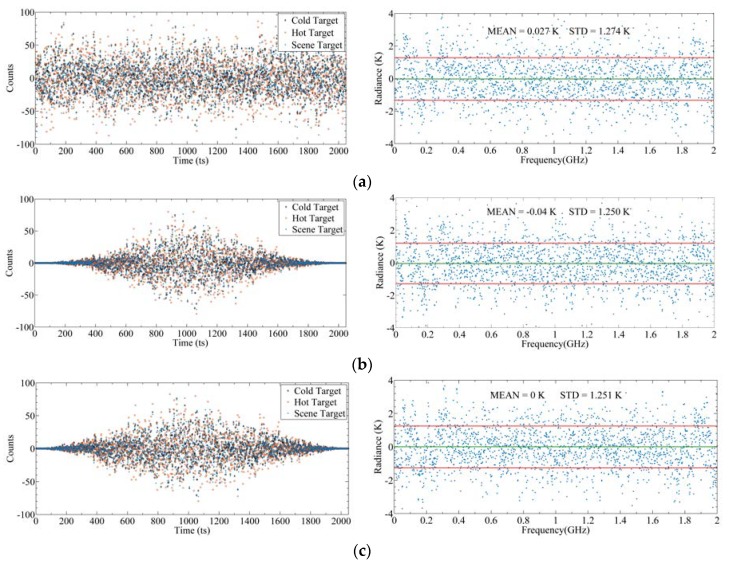
Backend signals and corresponding sensitivities with different window functions: (**a**) Rectangular window; (**b**) Hanning window; (**c**) Blackman window. The left figures represent output signals after quantization, and the right figures represent corresponding differences between input radiance and output radiance.

Figure 19 shows the backend signal and corresponding sensitivity with different window functions. It can be seen that the sensitivities calculated by the three window functions are at the same level. The sensitivity of the rectangular window is 1.274 K, which is slightly worse than that of the Hanning window and Blackman window. This may be due to the fact that compared with main lobe broadening, the influence of spectrum leakage on the FFT operation of the noise signal is more serious. The rectangular window has the worst suppression effect on spectrum leakage, so the spectrum resolution is the poorest. The sensitivity results calculated by the Blackman window and Hanning window are basically the same. In radio astronomy applications, the Blackman window has a good capability of spectrum leakage attenuation and amplitude retention of the random noise input signal [33]. Therefore, we chose the Blackman window as the window function.

### 4.4. Quantization Error

Quantization is the process of analog-to-digital conversion for the frontend output signal. The radiometer needs to sample the signal uniformly and continuously, so uniform sampling is adopted. The quantization process will inevitably lead to a decline of the measurement accuracy [34]. We can define the quantitative deterioration ratio η as

(13) $$\eta =\frac{{\left(\Delta R\right)}_{dig}}{{\left(\Delta R\right)}_{ideal}},$$

where ${\left(\Delta R\right)}_{dig}$ is the sensitivity after digital quantization and ${\left(\Delta R\right)}_{ideal}$ is the ideal sensitivity. The traditional radiometer quantization error has been discussed a lot, and the conclusion is clear, that is, at 3-bit, η is about 1.05. However, there has been little discussion about the influence of quantization error on spectrometers. The quantization error of autocorrelation spectrometers is presented in [35], and the quantization deterioration ratio is about 1.235 at 3-bit and 1.205 at 5-bit. We analyzed the quantization error of the FFT spectrometer by the TALIS simulation model. The quantization process was completed by Equation (6).

**Figure 20:**
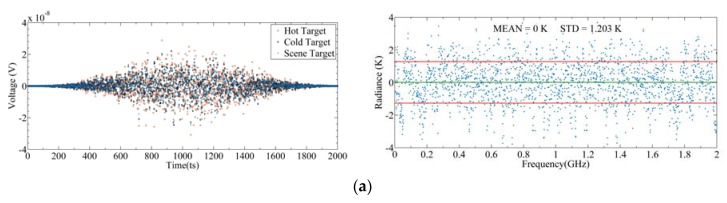

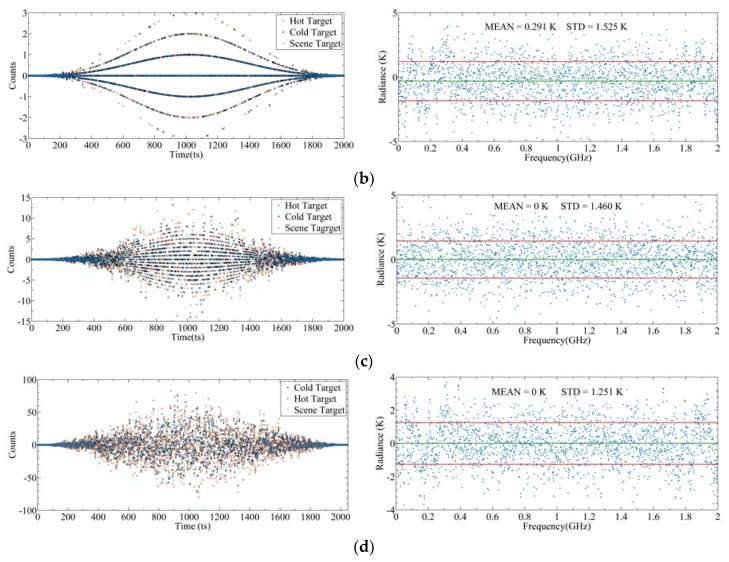
Backend signals under different quantization levels and corresponding sensitivities: (**a**) Analog; (**b**) 3-bit; (**c**) 5-bit; (**d**) 8-bit. The left figures represent output signals after quantization, and the right figures represent corresponding differences between input radiance and output radiance.

Figure 20 shows backend signals under different quantization bits and their corresponding sensitivities. The quantization levels are 3-, 5-, and 8-bit, respectively, and the spectrum resolution and integration time are 2 MHz and 100 ms, respectively. To validate the simulation model, we simulated the quantization error of the total power radiometer, that is, using a detector model to replace the spectrometer model in the backend [28]. The simulation results show that the deterioration ratio of simulation sensitivity and the theoretical sensitivity of the total power radiometer are almost the same. At 3-bit, the deterioration ratio is 1.065 and 1.053, respectively, which verifies the simulation model. As for the spectrometer, the deterioration ratio is 1.267 at 3-bit. Compared with the total power radiometer, the deterioration ratio of the spectrometer is significantly enlarged. This is because, according to the sensitivity equation, when the bandwidth is smaller, the signal fluctuation is more intense. The sensitivity of the total power radiometer is the result of the whole band, while the sensitivity of the spectrometer is only the result of one channel, so the deterioration would be higher. A comparison of η from the FFT spectrometer, calculated by the TALIS simulation model, and the autocorrelation spectrometer, given by [35], is shown in Table 3. At 3- and 5-bit, the η values of the two digital spectrometers are basically the same. For the TALIS spectrometer, 8-bit quantization with the sensitivity of 1.251 K is good enough to meet the sensitivity requirements.

## 5. Conclusions

This paper introduces the preliminary simulation of the instrument performance of TALIS. At first, we established the simulation model for TALIS, and performed a joint simulation with ARTS. The TALIS simulation model is divided into three parts, which are the input signal model, RF frontend model, and digital backend model. The input signal model is based on WGN to build the thermal noise signal with the radiance spectrum, the RF frontend model describes the system transfer function through two sideband SRFs, and the backend model uses the FFT algorithm to calculate the power spectrum to describe the working process of the digital spectrometer. The performance was evaluated from the aspects of calibration, the imbalance of two DSB sidebands, the spectrum resolution, and quantization error. The simulation results show that two-point calibration can eliminate the influence of SRF and obtain the scene target radiance spectrum. In a DSB receiver, although some lines will be weakened or overlapped, most lines are retained. Due to the wide bandwidth, the sensitivity of DSB is better. The sideband imbalance of 0.5% will cause a maximum radiance error of 0.6 K for 240 GHz S5. Therefore, the sideband imbalance of TALIS should be better than 0.5%. The results show that when the number of channels is 1024, the spectrum resolution and sensitivity can meet the requirements simultaneously. The quantization also has an influence on the instrument sensitivity. When the quantization level is 8-bit, the sensitivity and sensitivity deterioration ratio are 1.251 K and 1.039 with a 2 MHz spectrum resolution and a 100 ms integration time, respectively, which can meet the requirements.

The research results of this paper are helpful for preliminary evaluating the instrument performance of TALIS in the system design stage. The analysis results can provide guidance for the parameter design of TALIS. For example, through the TALIS simulation model, the output results of the current indicator can be estimated. In addition, when the actual performance of the instrument does not meet the expectations, the possible sources of error can be analyzed through the model, etc. It is undeniable that the model proposed in this paper is still simple, for example, the antenna and gain fluctuation are not considered, and future work will focus on solving these problems to make the model more consistent with the real instrument.

## Figures and Tables

**Table 1:** Characteristics of the THz atmospheric limb sounder (TALIS) instrument.

Band (Local Oscillator (LO)) (GHz)	*Tsys* (K)	Spectrometer	Band (GHz)	Sensitivity (K)	Main Products
118 (117.55)	1000	S1	115.35–117.35 117.75–119.75	2.2	O_2_
190 (190.10)	1000	S2	175.5–177.5 202.7–204.7	2.2	O_3_ HCN
		S3	178.9–180.9 199.3–201.3		N_2_O ClO
		S4	183.0–185.0 195.2–197.2		O_3_ H_2_O
240 (239.66)	1000	S5	229.66–231.66 247.66–249.66	2.2	O_3_ CO
		S6	232.16–234.16 245.16–247.16		O_3_ O_2_
		S7	234.66–236.66 242.66–244.66		O_3_
643 (642.87)	2300	S8	624.47–626.47 659.27–661.27	5.1	ClO BrO HO_2_
		S9	627.37–629.37 656.37–658.37		HNO_3_
		S10	632.37–634.37 651.37–653.37		N_2_O
		S11	624.47–626.47 659.27–661.27		HCl BrO

**Table 2:** Means and standard deviations of radiance difference at different integration times.

Integration Time (ms)	Theoretical Value (K)		Simulated Value (K)	
	Means	STD	Means	STD
1	0	11.18	0.018	11.82
10	0	3.54	0.027	3.87
100	0	1.12	−0.015	1.25

**Table 3:** Deterioration ratio η under different quantization levels.

Quantization Level	FFT Spectrometer	Autocorrelation Spectrometer
3	1.267	1.235
5	1.213	1.205
8	1.039	-
